# Multifunctional Zinc Glycyrrhizinate‐Integrated Gel for Enhanced Probiotic Delivery and Alleviation of Acute and Spontaneous Colitis

**DOI:** 10.1002/advs.77674

**Published:** 2026-09-13

**Authors:** Mengyun Peng, Xiaochun Chen, Wei Li, Shu Tang, Jun Zhou, Xia Wu, Dan Yuan, Zhongyan Shan, Junfeng Shi

**Affiliations:** ^1^ Hunan Provincial Key Laboratory of Animal Models and Molecular Medicine State Key Laboratory of Chemo/Bio‐Sensing and Chemometrics School of Biomedical Sciences Hunan University Changsha Hunan China; ^2^ Changsha Stomatological Hospital Changsha Hunan China; ^3^ School of Stomatology Hunan University of Chinese Medicine Changsha Hunan China; ^4^ Shenzhen Research Institute Hunan University Shenzhen Guangdong China

**Keywords:** drug repurposing, gut microbiota, IL‐10‐deficient, Licorzinc, probiotics delivery

## Abstract

Effective probiotic therapy for colitis is hindered by the harsh gastrointestinal (GI) physiologic and pathologic environment, including acidic pH, bile salts, elevated reactive oxygen species (ROS), mucus depletion, and dysbiosis. To address these challenges, we developed a novel “drug carrier‐integrated” oral probiotic delivery system—*L. reuteri*@ZGM—based on a zinc glycyrrhizinate microgel (ZGM) derived from the clinical anti‐inflammatory agent Licorzinc. This multifunctional gel encapsulates *L. reuteri* within a porous network, achieving a 23.08‐fold increase in survival rate under simulated gastric conditions and a 37.22‐fold increase in intestinal colonization in mice compared to unencapsulated bacteria. Beyond protection, ZGM actively scavenges ROS at inflammatory sites, facilitates probiotic adhesion to the mucus layer, and modulates the local microenvironment to favor colonization. In both dextran sulfate sodium (DSS)‐induced colitis and IL‐10‐deficient spontaneous colitis mouse models, *L. reuteri*@ZGM persistently scavenged ROS, effectively reduced pro‐inflammatory cytokine levels, restored the expression of tight junction‐associated proteins and intestinal barrier function, enhanced probiotic colonization and survival, and reshaped the gut microbiota. These findings establish *L. reuteri*@ZGM as a potent therapeutic strategy that integrates microenvironment modulation and probiotic delivery—offering a clinically translatable approach to treating inflammatory bowel diseases.

## Graphical abstract

Multifunctional oral delivery system—*L. reuteri*@ZGM—act as both a carrier and therapeutic adjuvant, providing ROS scavenging, and mucin‐interactive properties tailored to the IBD microenvironment. This bioactive gel safeguards *L. reuteri* from gastric degradation, enhances mucosal adhesion, and promotes long‐term retention at inflamed sites, thereby facilitating sustained microbiota modulation and mucosal repair. Furthermore, *L. reuteri*@ZGM systematically reversed colonic inflammation in DSS‐induced colitis and *IL‐10* ^−/−^ spontaneous mouse colitis models, by scavenging ROS, inhibiting pro‐inflammatory cytokines production, restoring intestinal barrier integrity.

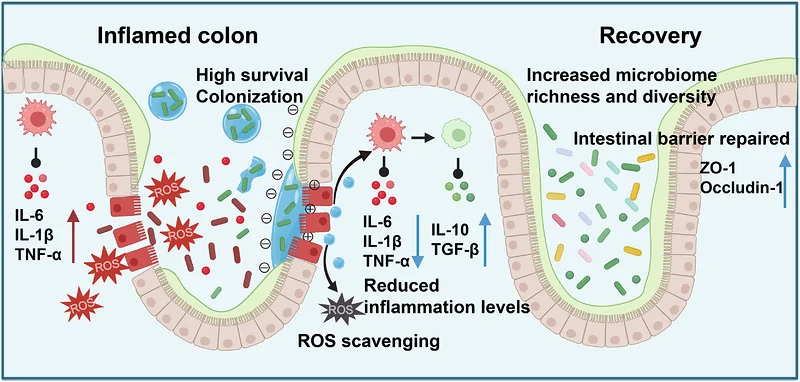

## 1 Introduction

Inflammatory bowel disease (IBD), encompassing Crohn's disease (CD) and ulcerative colitis (UC), is a chronic, relapsing condition characterized by persistent inflammation of the GI tract [1]. Often referred to as the “green cancer,” IBD places a substantial health and economic burden on global societies, severely impairing the quality of life for affected individuals [2]. The pathogenesis of IBD remains complex and multifactorial, with recent evidence implicating a combination of intestinal mucosal barrier dysfunction [3], gut microbial disorders [4], and an excessive immune response driven by elevated ROS and inflammatory factors [5, 6]. Current pharmacotherapies, including the anti‐inflammatory drug 5‐aminosalicylate (5‐ASA), corticosteroids, and tumor necrosis factor (TNF) antagonists, focus on mitigating the inflammatory response [7]. However, these drugs are also frequently associated with systemic side effects, including immunosuppression and long‐term complications, and may necessitate adjunctive antibiotic use in certain clinical settings. Moreover, they provide only symptomatic relief, failing to address the underlying disruptions in the intestinal barrier and microbiome [8].

Oral probiotic therapy has emerged as a promising alternative approach due to its potential to restore intestinal flora balance [9, 10], enhance mucosal healing [11], and modulate host immunity [12, 13, 14]. Certain strains, particularly *Lactobacillus* and *Bifidobacterium* species, have been shown to maintain intestinal barrier function and alleviate inflammation in IBD [15]. However, the clinical success of probiotics is limited by their inadequate survival in the harsh GI environment, and poor retention during transit through the stomach and small intestine [16, 17]. The GI tract's acidic pH, bile salts, and mechanical peristalsis all pose significant challenges to the effective delivery of probiotics, reducing their therapeutic potential [18]. Recent advances in drug delivery technologies have focused on addressing these barriers by encapsulating probiotics within protective carriers such as protective layer [19], liposomes [20], polymer gels [21], bacterial biofilms [22], and cell membranes [23]. These strategies aim to shield probiotics from digestive stress and enhance their stability, but they typically focus on protecting probiotics from physiological conditions rather than pathological factors inherent in IBD [24]. In particular, elevated ROS levels at the site of inflammation can damage probiotic cell membranes, leading to inactivation and impaired colonization [25]. Furthermore, the depletion of the mucus layer in inflamed tissues further hinders bacterial adhesion and persistence [26, 27]. Thus, there is a critical need for probiotic delivery systems that are specifically tailored to resist both the physiological and pathological challenges posed by IBD.

**SCHEME 1:**
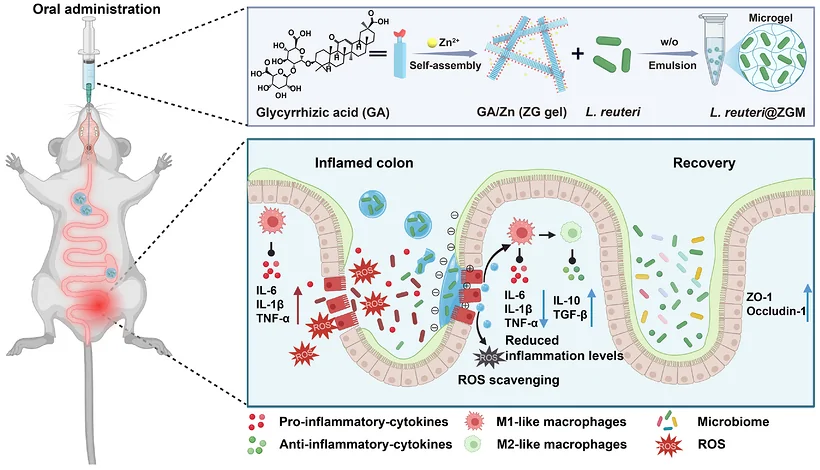
Illustration of *L.reuteri*@ZGM mechanisms in IBD treatment. *L. reuteri*@ZGM systematically reversed colonic inflammation by scavenging ROS, inhibiting the production of pro‐inflammatory cytokines, restoring intestinal barrier function, and increasing the abundance and diversity of the intestinal microbiota.

In this study, we report a multifunctional oral delivery system—*L. reuteri*@ZGM—based on a zinc‐crosslinked glycyrrhizinate microgel (ZGM) self‐assembled from glycyrrhetinic acid (GA), the hydrolyzed product of GA that is the main component of the clinical anti‐inflammatory drug, Licorzinc [28]. GA is a naturally derived triterpenoid saponins with established anti‐inflammatory, antioxidant, and mucosa‐protective properties via inhibition of NF‐κB signaling [29]. By incorporating GA into a Zn^2^^+^‐coordinated microgel, we designed ZGM to act as both a carrier and therapeutic adjuvant, providing ROS scavenging, antimicrobial, and mucin‐interactive properties tailored to the IBD microenvironment. This bioactive gel safeguards *L. reuteri* from gastric degradation, enhances mucosal adhesion, and promotes long‐term retention at inflamed sites, thereby facilitating sustained microbiota modulation and mucosal repair (Scheme 1). Furthermore, *L. reuteri*@ZGM systematically reversed colonic inflammation in DSS‐induced colitis and *IL‐10*‐deficient (*IL‐10^−/−^*) mice with spontaneous colitis models, by scavenging ROS, inhibiting pro‐inflammatory cytokines production, restoring intestinal barrier integrity, leading to improved microbiome diversity and abundance. Therefore, the drug derivative gel *L. reuteri@ZGM* establishes a broader paradigm for repurposing natural‐product‐derived agents as dual‐function therapeutic carriers in inflammatory disease management.

## 2 Results and Discussion

### 2.1 Preparation and Characterization of ZG Gel

**FIGURE 1:**
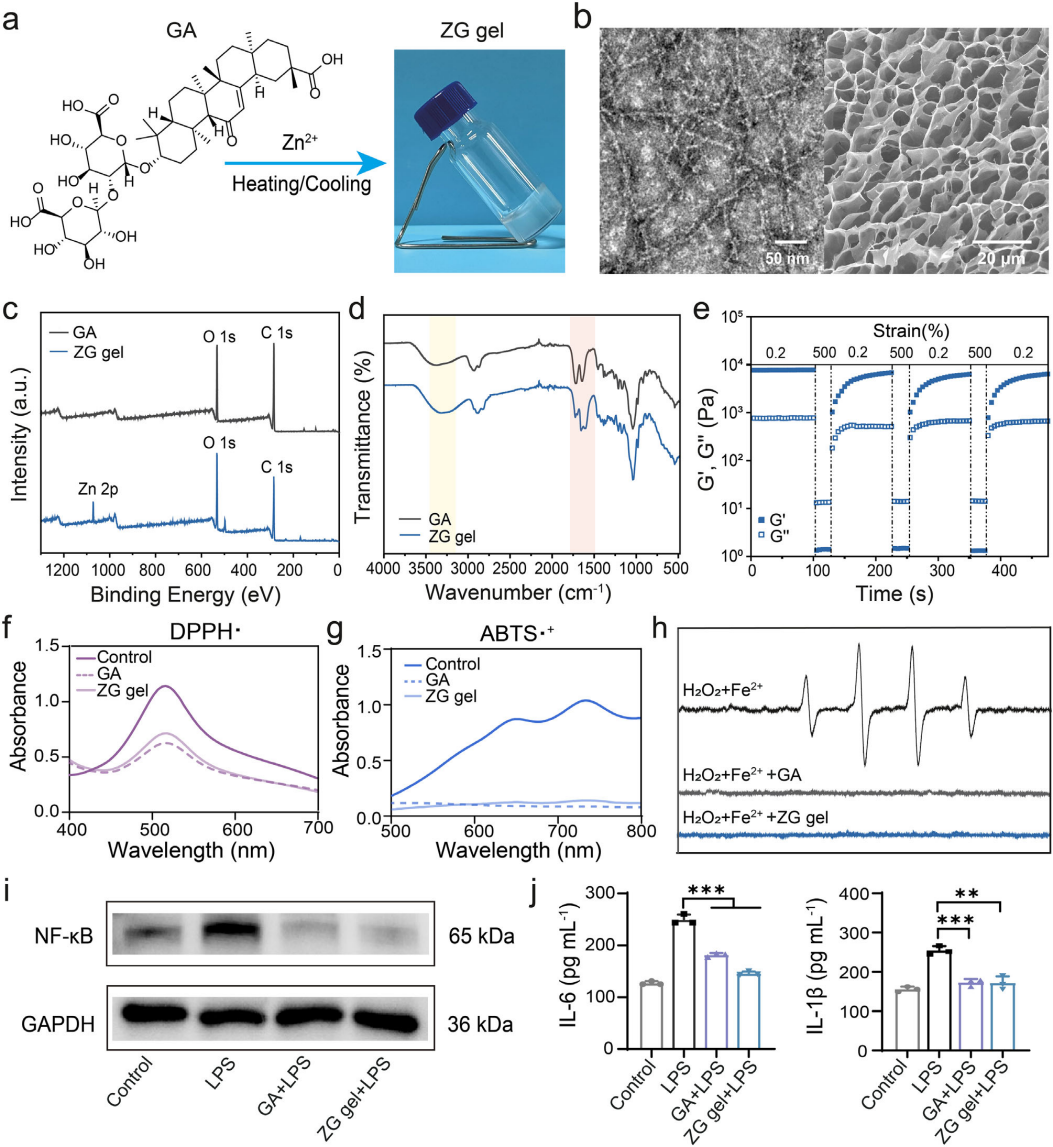
Biophysical characterization of ZG gel. a) Schmatic of Hydrogel formation via a simple heating‐cooling method, where a mixed solution of GA and ZnSO_4_ is heated to 80°C and subsequently cooled to room temperature to trigger gelation. b) TEM (right) and SEM (left) images of the ZG gel, with a scale bar of 50 nm (TEM) and 20 µm (SEM). c‐d) XPS and FTIR spectra of the ZG gel, demonstrating its elemental composition and structural characteristics. e) Rheological measurement showing the recovery of storage modulus (G') during strain cycles ranging from 0.2% (low strain) to 500% (high strain), at a frequency of 1 Hz. f‐h) Antioxidant activity of the ZG gel, assessed by its ability to scavenge free radicals (ABTS·^+^, DPPH·, and ·OH). i‐j) Relative expression of NF‐κB, IL‐6 and IL‐1β in RAW 264.7 cells exposed to LPS, LPS + GA and LPS + ZG gel (n = 3). Data represent geometric means ± SEM, with *p* values determined by Student's *t‐test*. *p < 0.05, **p < 0.01, and ***p < 0.001.

The zinc glycyrrhizinate gel (ZG gel) was prepared using a simple heating‐cooling method (Figure 1a). Transmission electron microscopy (TEM) image revealed that ZG self‐assembled to form nanofibers‐rich network [30]. Meanwhile, scanning electron microscopy (SEM) image display a porous structure within the ZG gel matrix (Figure 1b). The elemental composition of ZG gel was investigated using X‐ray photoelectron spectroscopy (XPS). The spectra measured from 0 to 1200 eV, confirmed the presence of carbon (C), oxygen (O), and zinc (Zn) as the major components of the gel (Figure 1c). High‐resolution XPS analysis of Zn2p and O1s regions further revealed metal‐ligand bonding between Zn^2+^ and carboxylates from GA in the gels (Figure S1). In addition, the ζ‐potential of the ZG gels, measured by dynamic light scattering, was ‐14.1 ± 0.7 mV (Figure S2), indicating the involvement of Zn^2+^ in the gel network. Fourier transform infrared (FT‐IR) spectroscopy analysis (Figure 1d) demonstrated structural changes after gelation. Specifically, the O─H tensile vibration peak at 3373 cm^−1^ shifted to 3301 cm^−1^ with increased intensity, and the C═O stretching vibration in the carboxyl group shifted from 1724 and 1644 cm^−1^ to 1659 and 1595 cm^−1^, respectively, confirming the hydrogen bonding interactions during gel formation [31]. Finally, to assess the rheological properties of the ZG gel, oscillation tests were performed. At low strain (0.2%), the gel exhibited a higher G' (storage modulus) than G“ (loss modulus), indicating its gel state [32]. At high strain (500%), G' decreased below G”, suggesting the disruption of the hydrogel network. Upon restoring low strain, the gel's stiffness recovered, demonstrating its self‐healing characteristics (Figure 1e; Figure S3). This self‐healing property is crucial for maintaining structural integrity and adhesive persistence under dynamic mechanical stresses such as intestinal peristalsis, forming the basis for its excellent gastrointestinal adaptability.

### 2.2 Biological Properties of ZG Gel

Next, we investigated the biological properties of ZG gel. Biocompatibility is the key consideration for the application of materials in the biomedical field. To assess this, cytotoxicity tests were performed using Caco‐2 intestinal epithelial cells. The results showed no significant change in cell viability after co‐culturing Caco‐2 cells with various concentrations of ZG gel for 24 h, indicating good cytocompatibility of the gel (Figure S4). To test whether the hydrogel can release probiotics under GI conditions, ZG gel was exposed to buffer at pH 1.2, 6.8, and 7.4. The gel maintained integrity for up to 24 h at pH 1.2 but began dissolving within 2 h at pH 7.4 (Figure S5). This pH‐responsive dissolution ensures that encapsulated probiotics are protected in the acidic stomach yet released in the small intestine and colon, enabling targeted delivery and optimized gastrointestinal compatibility. Given the high concentration of ROS in the pathological microenvironment of colitis, which can damage the probiotic cell wall and inactivate the bacteria, we tested the antioxidant capacity of ZG gel. Since GA contains two glucuronic acids, it is capable of scavenging ROS. We used three distinct assays—1,1‐diphenyl‐2‐picratehydrazyl (DPPH), 2,2'‐azido‐bis (3‐ethylbenzothiazole‐6‐sulfonic acid) diammonium salts (ABTS), and electron paramagnetic resonance (EPR) spectroscopy—to systematically evaluate the gel's antioxidant activity (Figure 1f–h) [33]. The ZG gel effectively scavenge DPPH·, ABTS·^+,^ and ·OH free radicals, demonstrating its excellent antioxidant capacity. To investigate the anti‐inflammatory mechanism of ZG gel, we examined its effects on RAW 264.7 macrophages stimulated by lipopolysaccharide (LPS) or IL‐10. Flow cytometry analysis revealed that, compared with the LPS and IL‐10 group, ZG gel inhibited M1‐like polarization and promoted M2‐like polarization (Figure S6). Western blot analysis revealed that LPS‐induced upregulation of NF‐κB protein expression was significantly inhibited by ZG gel (Figure 1i; Figure S7). Consistent with this, ELISA results indicated that in LPS‐treated cells, ZG gel significantly reduced the secretion of pro‐inflammatory cytokines IL‐6 and IL‐1β (Figure 1j). Therefore, ZG gel exerts its anti‐inflammatory effects by promoting M2 polarization, inhibiting the NF‐κB signaling pathway, and reducing inflammatory cytokine levels [34]. The ZG gel also exhibited strong tissue adhesion, with a maximum adhesion weight of 23 g on porcine skin (Figure S8), likely resulting from condensation reactions between its carboxyl and hydroxyl groups and amino groups on tissue surfaces [21]. Its moderate negative ζ‐potential promotes electrostatic interactions with the mucosal layer, while self‐healing rheology maintains structural integrity under peristaltic forces. Together, these properties enhance mucosal retention and support probiotic colonization.

In combination, these physicochemical properties—pH responsiveness, self‐healing rheology, ζ‐potential, and tissue adhesion—synergistically enable ZG gel to protect probiotics, adhere to the intestinal mucosa, and promote colonization, making it an ideal carrier for oral probiotic therapy in colitis.

### 2.3 Probiotics Encapsulation in ZG Gel and its Protective Effect

**FIGURE 2:**
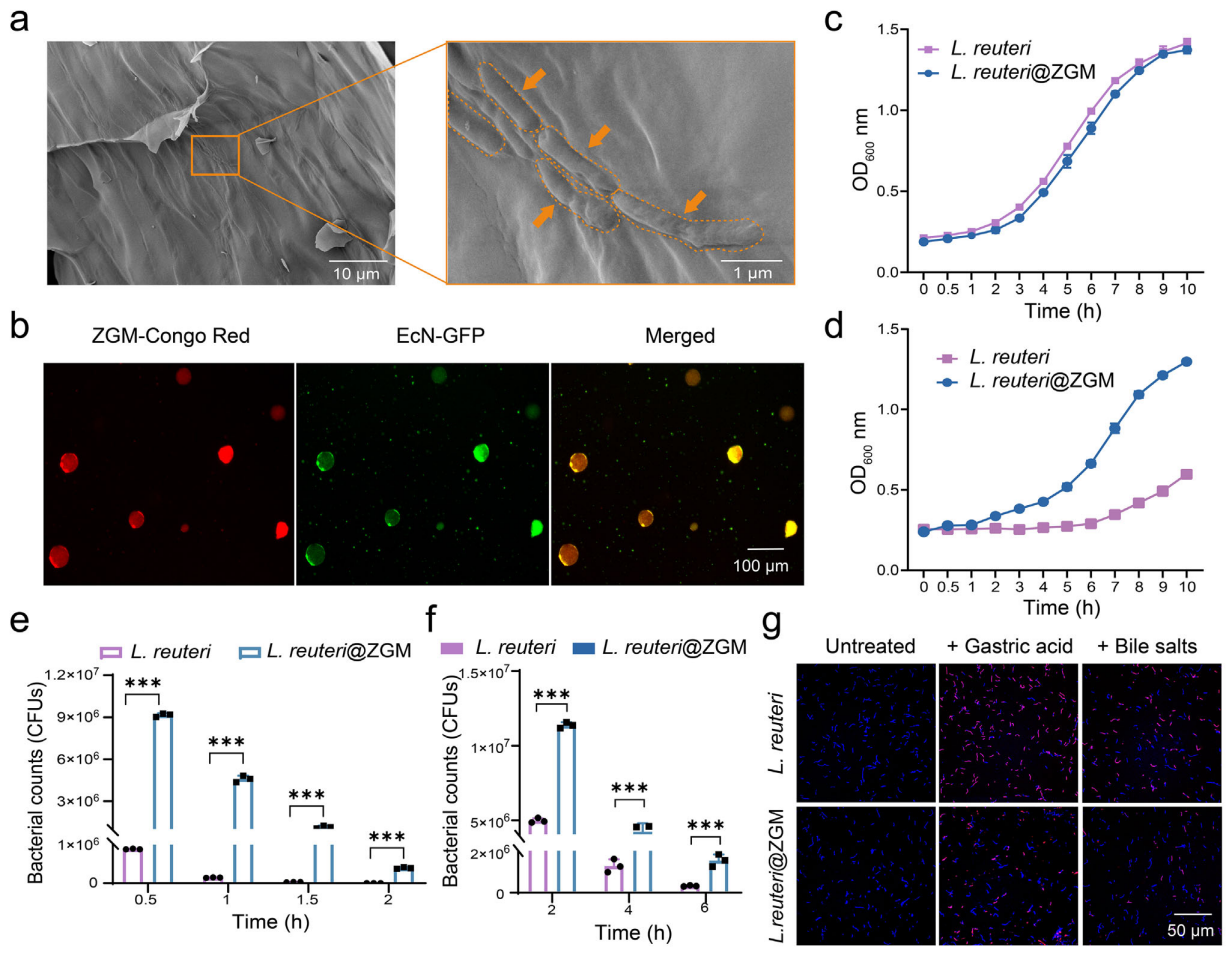
Encapsulation and protection of probiotics by ZGM. a) SEM images showing the distribution of *L. reuteri* within ZGM. b) The fluorescently modified probiotic EcN‐GFP encapsulated in ZGM. c) Growth curves of *L. reuteri* and *L. reuteri*@ZGM in MRS medium at 37°C, recorded every hour using a microplate spectrophotometer (n = 3). d) Growth curves of *L. reuteri* and *L. reuteri*@ZGM in MRS medium after treatment with simulated gastric fluid (SGF) and bile salts (n = 3). e‐f) Viability of equal amounts of *L. reuteri* and *L. reuteri*@ZGM exposed to SGF (e) and bile salts (f) (0.3 mg mL^−1^) at 37°C (n = 3). g) Live/dead cell viability assay of *L. reuteri* and *L. reuteri*@ZGM before and after treatment with SGF (0.5 h) and bile salts (1 h). Red indicates dead cells. Data represent geometric means ± SEM, with *p* values determined by Student's *t‐test*. *p < 0.05, **p < 0.01, and ***p < 0.001.

Zinc glycyrrhizinate microspheres (ZGM) encapsulating *L. reuteri* (*L. reuteri*@ZGM) were prepared using a double emulsification method, achieving a 94% encapsulation efficiency [35]. The encapsulated probiotics were clear observed in the SEM images (Figure 2a). Confocal laser scanning microscopy (CLSM) further confirmed the uniform and efficient encapsulation of probiotics within the ZGM, with a diameter size of approximately 82 µm (Figure 2b; Figure S9). Moreover, the growth rate of *L. reuteri* in the *L. reuteri*@ZGM group was comparable to that of unencapsulated *L. reuteri* growth in MRS Medium at 37°C, indicating that ZGM did not interfere with the activity or proliferation of *L. reuteri* (Figure 2c). The first challenge faced by probiotics after oral administration is the harsh physiological environment of the GI tract. We examined the viability of probiotics after exposure to GI components. The growth curve showed that *L. reuteri*@ZGM exhibited faster growth over time compared to free *L. reuteri*, indicating that ZGM protected *L. reuteri* from gastric acid and bile salt exposure, thereby preventing bacterial death (Figure 2d). In addition, bacterial plate counts confirmed that ZGM effectively shielded *L. reuteri* from gastric acid and bile salts. After 2 h of incubation in simulated gastric fluid (SGF), large number of viable cells were still observed in *L. reuteri*@ZGM group, while the uncoated *L. reuteri* group showed almost complete mortality (Figure 2e). As shown in Figure 2f, after 2 h of incubation with bile salts (0.3 mg mL^−1^), the viability of the free *L. reuteri* decreased significantly. Even after extending the incubation period to 6 h, the number of viable bacteria in the *L. reuteri*@ZGM group remained nearly 6‐fold higher (10^6^ CFUs mL^−1^) than in the uncoated *L. reuteri* group. The protective effect of ZG gel on probiotics was further confirmed by live/dead cell staining. A higher number of red dead cells were observed in the free *L. reuteri* group, whereas the majority of *L. reuteri*@ZGM cells remained viable (Figure 2g). These results conclusively demonstrate the successful encapsulation of *L. reuteri* by ZGM and its effective protection under GI conditions.

### 2.4 Enhanced Delivery, Survival and Colonization of *L. Reuteri* by ZGM In Vivo

**FIGURE 3:**
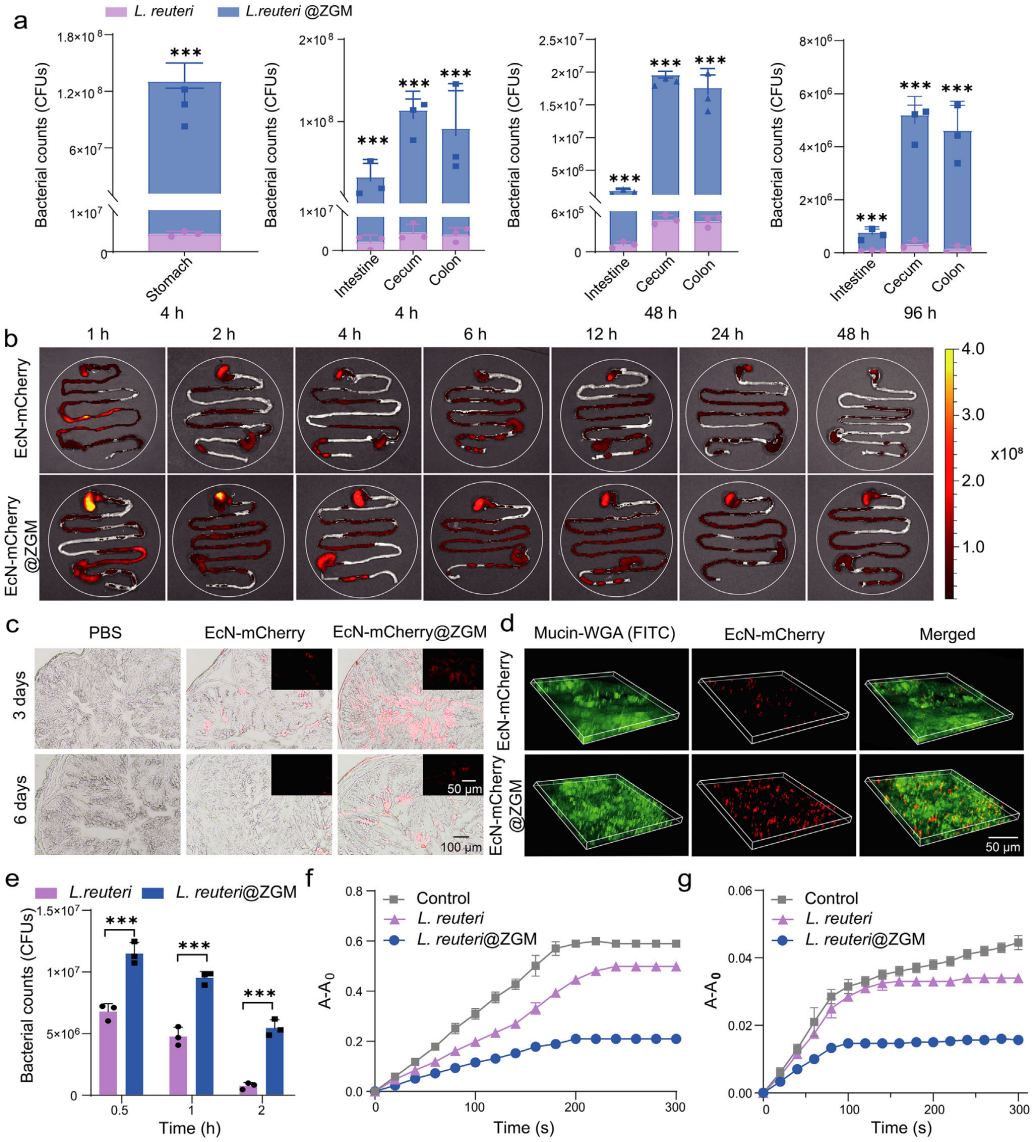
Enhanced delivery, survival and colonization of L. reuteri@ZGM in vivo. a) Survival of bacteria in the stomach 4 h after oral administration, and the number of bacteria in the intestine, colon, and cecum at 4, 48, and 96 h after administration, respectively. b) Distribution of EcN‐mCherry and EcN‐mCherry@ZGM in the GI tract within 48 h after oral administration, assessed by an intestinal transit assay. c) Long‐term intestinal adhesion of free EcN‐mCherry (red) and EcN‐mCherry@ZGM in vivo. Representative images of colonic sections from days 3 and 6 after a single gavage, obtained by CLSM. d) 3D images of free EcN‐mCherry and EcN‐mCherry@ZGM adhesion to intestinal mucus of mice. Red: EcN‐mCherry. Green: mucus stained with FITC‐WGA. Scale bar, 50 µm. e) Equal amounts of *L. reuteri* and *L. reuteri*@ZGM were exposed to H_2_O_2_ (500 µM) at the specified time points. After incubation at 37°C, 50 µL of each sample was washed with PBS, spread on the MRS agar plates, incubated for 12 h at 37°C, and the colonies were counted (n = 3). f) Kinetic curves of A‐A_0_ (415 nm) monitoring the reduction of ABTS with FeCl_2_ and H_2_O_2_ in the absence and presence of *L. reuteri* and *L. reuteri*@ZGM. g) Kinetic curves of A‐A_0_ (340 nm) monitoring the reduction of pyrogallol autoxidation in the absence and presence of *L. reuteri* and *L. reuteri*@ZGM. Data represent geometric means ± SEM, with *p* values determined by Student's *t‐test*. *p < 0.05, **p < 0.01, and ***p < 0.001.

To determine whether multifunctional gels can enhance probiotic delivery, survival, and colonization in the gut, *L. reuteri*@ZGM (1.0 × 10^9^ CFUs) was administered via gavage, and *L. reuteri* in the GI tract was quantified at various time points. The amount of *L. reuteri* in the stomach, small intestine, cecum, and colon was determined by plate colony counting. As shown in Figure 3a, the *L. reuteri*@ZGM group far exceeded the number of *L. reuteri* group in all regions examined. Interestingly, at 4 h post‐administration, the *L. reuteri*@ZGM group had 23.08‐fold more viable bacteria in the stomach compared to *L. reuteri* group. Notably, *L. reuteri*@ZGM showed significant adherence to the colonic mucosa, with 18.9‐fold higher than *L. reuteri* group. Similar results were obtained at 48 and 96 h, with 37.22‐fold and 24.4‐fold higher colonization, respectively, compared to *L. reuteri* group. Furthermore, the total number of viable *L. reuteri*@ZGM in the GI tract was quantified, confirming significantly higher survival and colonization even at 96 h. The *L. reuteri*@ZGM group had substantially greater colonization in the small intestine (p < 0.001), cecum (p < 0.001), and colon (p < 0.001) relative to the *L. reuteri* group (Figure S10). Taken together, these results demonstrate that multifunctional gels can effectively enhance the delivery, survival, and colonization of probiotics in the gut.

Since the long‐term retention of probiotics in the gut is critical for the successful establishment of colonies, we further analyzed the retention and distribution of probiotics in the gut using an in vivo imaging system. EcN‐mCherry expressing red fluorescence was encapsulated by ZGM (EcN‐mCherry@ZGM) for quantitative analysis. As shown in Figure 3b and Figure S11 (Supporting Information), free EcN‐mCherry was unstable in the stomach and weakly interacted with intestinal mucus, and essentially disappeared after 48 h. Interestingly, substantial fluorescence could still be detected at EcN‐mCherry@ZGM, indicating enhanced retention and adhesion of EcN‐mCherry@ZGM. In addition, the fluorescence signal of other major organs (heart, liver, spleen, lung, and kidney) was negligible throughout the assay period, indicating that EcN‐mCherry@ZGM was metabolized in the GI tract following gastric administration (Figure S12). To observe long‐term intestinal adhesion of capsule probiotics, we examined the presence of probiotics in the gut on days 3 and 6 after a single oral administration of 1 × 10^9^ CFUs of EcN‐mCherry and EcN‐mCherry@ZGM (Figure 3c). A weak fluorescent signal was observed in the EcN‐mCherry group on day 3, but no fluorescent signal was detected on day 6, suggesting that they were completely cleared from the intestine. Surprisingly, fluorescent signals were visible on days 3 and 6 in the EcN‐mCherry@ZGM treatment group, which strongly suggests that our designed multifunctional gel‐encapsulated probiotic system increases the retention time of EcN‐mCherry@ZGM throughout the gut by interacting with mucus. To visually confirm our hypothesis, we investigated the three‐dimensional adherence of probiotics to the colonic mucus layer (Figure 3d). The amount and intensity of EcN‐mCherry@ZGM fluorescence in mucus was much higher than that in the EcN‐mCherry group, indicating that multifunctional gel encapsulation greatly improved the adhesion of this probiotic to mucus.

In the inflammatory pathological microenvironment, various immune cells (neutrophils, leukocytes, inflammatory macrophages, etc.) are highly permeable, leading to a large increase of ROS in the gut [25]. ROS can reduce probiotic activity by oxidatively damaging lipids, proteins, and DNA, further disrupting probiotic colonization in the gut [36]. Therefore, we further evaluated the resistance of encapsulated probiotics to ROS. As shown in Figure 3e, almost all of the *L. reuteri* group died after incubation in H_2_O_2_ (500 µM) up to 2 h, while a large number of *L. reuteri*@ZGM survived. Bacterial viability was further assessed using CCK‐8. The results showed higher bacterial viability in the *L. reuteri*@ZGM group compared to the *L. reuteri* group after H_2_O_2_ treatment (Figure S13). This may be due to the antioxidant capacity of the multifunctional ZGM, which protects the probiotics from ROS damage. Then we tested the ability of *L. reuteri*@ZGM and *L. reuteri* to eliminate two other ROS (·OH and ·O^2−^) in vitro. First, ·OH produced by the Fenton reaction was detected by a specific ABTS probe, which can be oxidized by ·OH and produces a characteristic UV absorption peak at 415 nm. As shown in Figure 3f, the *L. reuteri*@ZGM group reduced the absorption intensity of ·OH compared with the control and *L. reuteri* groups. In addition, the auto‐oxidation of pyrogallol produces ·O^2−^, and the ability of *L. reuteri*@ZGM to eliminate ·O^2−^ was assessed by detecting the change in the UV absorption of ·O^2−^ at 340 nm for 5 min. *L. reuteri*@ZGM showed a strong ability to remove ·O^2−^ compared to the control and *L. reuteri* groups (Figure 3g). The above results suggest that *L. reuteri*@ZGM has a strong ability to adhere, retain, and remove ROS, which provides a firm foundation for the survival and colonization of oral probiotics in the gut.

### 2.5 *L. Reuteri*@ZGM Exerts a Powerful Therapeutic Effect in DSS Induced Colitis

**FIGURE 4:**
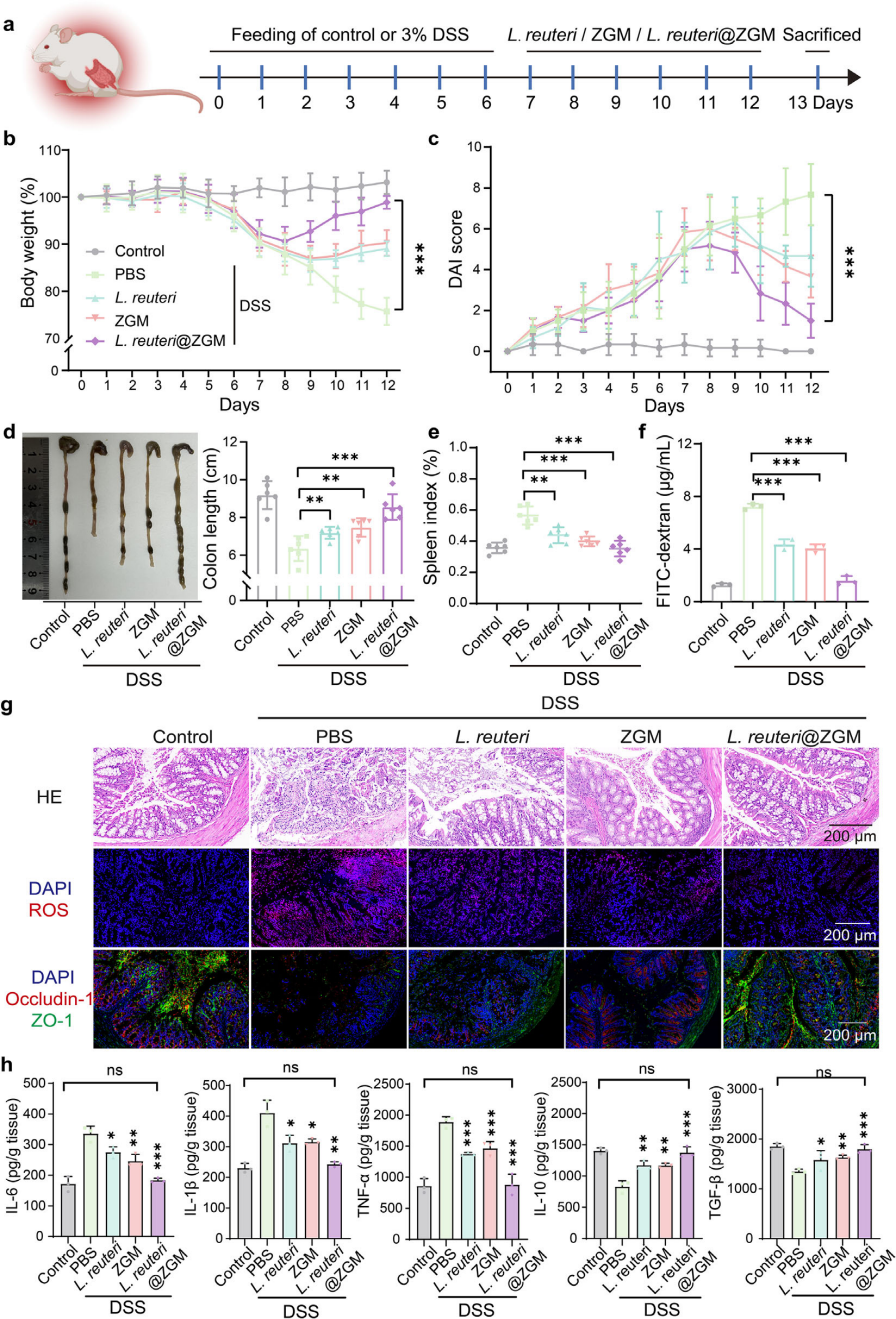
*L. reuteri*@ZGM exerts a powerful therapeutic effect in DSS‐induced colitis. a) Schematic representation of the experimental design for modeling and treatment. Mice were fed with water or water containing 3% DSS for 7 days, followed by daily oral treatment with *L. reuteri*, ZGM, *L. reuteri*@ZGM, or PBS for 5 days. On day 13, the mice were sacrificed for analysis. b) Body weight changes in mice across different treatment groups during the 13‐day period (n = 6). c) Disease Activity Index (DAI) scores for mice in each group (n = 6). d) Representative colonic tissue images and colon length measurement for mice in each group (n = 6). e) Spleen index measurement for mice on day 13 (n = 6). f) Evaluation of intestinal barrier function on day 13. Mice were orally administered with 4 kDa FITC‐dextran, and fluorescence intensity in serum was measured 4 h later. g) Representative images showing HE, ROS level, and ZO‐1/Occludin‐1 immunofluorescence staining in colon tissues. Scale bar, 200 µm. h) Analysis of pro‐inflammatory (IL‐6, IL‐1β, TNF‐α) and anti‐inflammatory (TGF‐β, IL‐10) cytokine levels in colonic tissues on day 13 (n = 3). Data represent geometric means ± SEM, with *p* values determined by Student's *t‐test*. *p < 0.05, **p < 0.01, and ***p < 0.001.

DSS‐induced colitis is a widely used animal model to simulate IBD [37]. We evaluated the therapeutic efficacy of *L. reuteri*@ZGM in treating DSS‐induced colitis in mice. Figure 4a depicts the experimental design for studying colitis in mice. Compared with the PBS, the *L. reuteri*@ZGM treated mice rapidly regained body weight, whereas those treated with ZGM and *L. reuteri* failed to prevent weight loss (Figure 4b). As shown in Figure 4c, the Disease Activity Index (DAI) quantifies the severity of colitis by scoring weight loss, stool consistency, and fecal bleeding (Table S1) [39]. The DAI values in the control group tended to stabilize, whereas those in the PBS‐treated group increased rapidly, indicating a more severe form of colitis. On day 13, the DAI score in the *L. reuteri*@ZGM treatment group was lower than that in the DSS treatment group, indicating that *L. reuteri*@ZGM is effective in alleviating the severity of colitis (Figure 4c). The length and condition of the colon serve as visual indicators of the severity of DSS‐induced colitis. In the colon photographs of the BC group, residual bloody stools were evident, whereas the condition improved significantly after *L. reuteri*@ZGM treatment. Furthermore, the colon length in the *L. reuteri*@ZGM treatment group was significantly longer than that in the ZGM group and the *L. reuteri* group, indicating that *L. reuteri*@ZGM protects colonic tissue from DSS‐induced damage (Figure 4d). Additionally, the spleen index was significantly reduced in the *L. reuteri*@ZGM treatment group, suggesting that the systemic inflammatory response had been suppressed (Figure 4e). Furthermore, in DSS‐induced colitis mice, the *L. reuteri*@ZGM‐treated group significantly reduced systemic exposure after oral administration of fluorescein isothiocyanate (FITC)‐dextran (*p* < 0.001, Figure 4f), indicating improved intestinal barrier integrity and reduced intestinal permeability. Immunofluorescence staining showed that administration of *L. reuteri*@ZGM normalized the expression levels of Occludin‐1 and ZO‐1, important components of intestinal epithelial tight junctions (Figure 4g). Alcian blue/periodic acid Schiff (AB/PAS) staining of the colon also showed that *L. reuteri*@ZGM treatment significantly restored the mucus layer (Figure S14). Additionally, hematoxylin‐eosin (HE) ‐staining revealed severe histological damage in the DSS‐treated group, including irregular epithelial structures, loss of crypts, and depletion of small intestinal cells (Figure 4g). In contrast, *L. reuteri*@ZGM treatment exhibited histological features similar to those of healthy group, with intact surface epithelium and crypt structure. Histopathological scores reflected these changes, with the DSS‐treated group exhibiting the highest scores, which were significantly reduced by *L. reuteri*@ZGM treatment (Figure S15). TUNEL staining further confirmed that *L. reuteri*@ZGM treatment effectively reduced DSS‐induced apoptosis in colon tissue (Figure S14). Moreover, ROS levels were significantly lower in the *L. reuteri*@ZGM‐treated group compared to the DSS, *L. reuteri*, and ZGM groups (Figure 4g). The reduced levels of ROS indicate that *L. reuteri*@ZGM can effectively regulate the intestinal microenvironment. In addition, pro‐inflammatory cytokines (IL‐6, IL‐1β, and TNF‐α) were significantly reduced in the *L. reuteri*@ZGM ‐treated group, while anti‐inflammatory cytokines (TGF‐β and IL‐10) were markedly increased (Figure 4h). Moreover, immunofluorescence analysis showed that *L. reuteri*@ZGM treatment reduced M1 macrophages (INOS^+^) while increasing M2 macrophages (CD206^+^) in colonic tissue (Figure S16). These results indicate that *L. reuteri*@ZGM promotes macrophage polarization toward an anti‐inflammatory M2 phenotype, thereby contributing to inflammation resolution and tissue repair. Finally, *L. reuteri*@ZGM treatment did not induce any overt signs of systemic toxicity in the major organs (Figure S17). It is important to note that in the DSS‐induced colitis model, epithelial injury and barrier dysfunction are largely driven by acute inflammatory stress and dysbiosis and remain partially reversible after DSS withdrawal. Upon cessation of DSS exposure, the intestinal epithelium retains a strong regenerative capacity, allowing rapid functional recovery when inflammatory stimuli are alleviated. Collectively, all these results demonstrated the superior efficacy of *L. reuteri*@ZGM in treating colitis.

### 2.6 Modulation of the Gut Bacterial Microbiota by *L. Reuteri*@ZGM in Colitis

**FIGURE 5:**
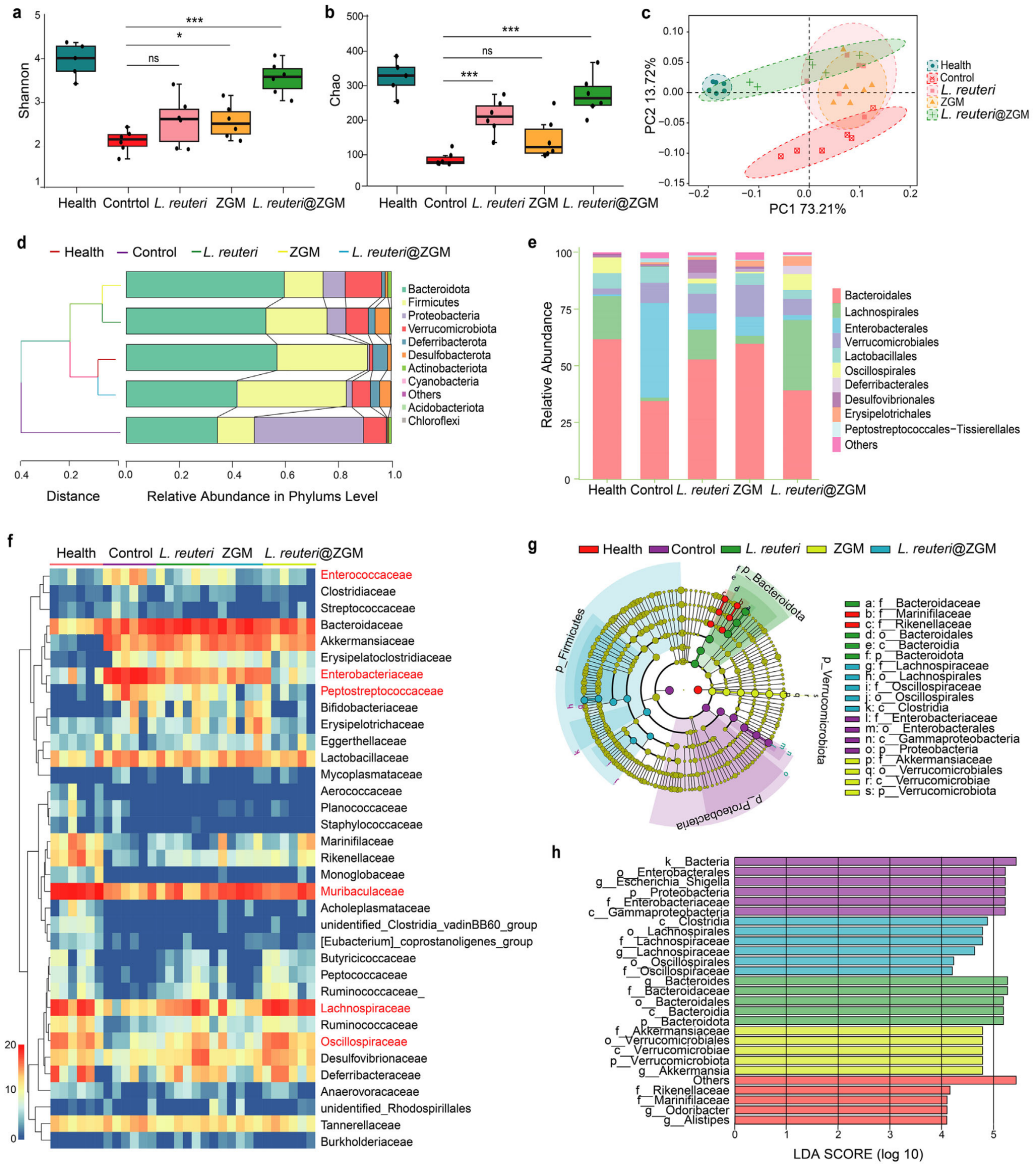
Modulation of the gut bacterial microbiota by *L. reuteri*@ZGM in DSS‐colitis. BALB/c mice were treated as described in Figure 4a, and fecal samples were collected on day 12 for 16S rDNA amplicon sequencing. a, b) The *α*‐diversity of the gut microbe, measured using the Shannon (a) and Chao (b) diversity index (n = 6). c) Principal coordinate analysis (PCoA) plot showing the *β*‐diversity of the gut microbe based on 1461 OTUs (n = 6). d) UPGMA clustering tree analysis of microbial communities. e) Relative abundance profiles of the gut bacterial microbiota at the order level across different treatment groups. f) Heatmap representing the relative abundance of the top 35 family in the gut bacterial microbiota. g) Cladogram from LEfSe analysis showing community composition of the gut microbiota in mice treated with different interventions. h) The LEfSe analysis identified the characteristic bacteria in different groups at the genus level (LDA score (log 10) > 4). Data represent geometric means ± SEM, with *p* values determined by Student's *t‐test*. *p < 0.05, **p < 0.01, and ***p < 0.001.

Emerging evidence suggests that gut bacterial microbiota homeostasis plays a pivotal role in managing IBDs [38]. To explore whether *L. reuteri*@ZGM treatment could restore gut bacterial microbiota balance and alleviate colitis, we analyzed fecal samples collected on day 12 from BALB/c mice treated as shown in Figure 4a. The samples were subjected to 16S rDNA amplicon sequencing targeting the V4 region. First, compared to other treatment groups, *L. reuteri*@ZGM treatment significantly increased *α*‐diversity (Shannon and Chao indices), indicating that it effectively reversed the DSS‐induced decline in the richness and diversity of the gut bacterial microbiota and restored the homeostasis of the gut bacterial microbiota (Figure 5a,b; Figure S18). *β*‐Diversity analysis further confirmed that *L. reuteri*@ZGM treatment gut bacterial microbiota structure of the mice overlapped highly with that of the healthy group in the principal coordinate analysis (PCoA) space, while being distinctly separated from the other treatment groups, suggesting that *L. reuteri*@ZGM restored gut bacterial microbiota homeostasis rather than merely modulating the bacterial microbiota composition (Figure 5c). Subsequently, we examined the gut bacterial microbiota abundance at different taxonomic levels. The relative abundances of the top 10 bacterial microbiota at the phylum level were calculated. In general, an increased ratio of Bacteroidetes to Firmicutes reflects gut bacterial microbiota dysbiosis. At the phylum level, *L. reuteri*@ZGM partially restored the Firmicutes/Bacteroidetes ratio (Figure 5d). Furthermore, at the order and family levels, *L. reuteri*@ZGM several bacterial families associated with short‐chain fatty acid production and intestinal homeostasis, including *Lachnospiraceae* [39], *Oscillospiraceae* [40], and *Muribaculaceae* families [41] (Figure 5e,f). The recovery of these bacterial communities is directly linked to the production of metabolites such as butyrate and propionate; the former provides energy to colonic epithelial cells and promotes the expression of tight junction proteins, while the latter alleviates immune damage by inhibiting CD8^+^ T‐cell activation [42]. At the same time, *L. reuteri*@ZGM significantly reduced the relative abundance of the Proteobacteria phylum, particularly opportunistic pathogens such as the Enterobacteriaceae family. This is highly consistent with the LEfSe analysis results—*Escherichia_Shigella* (LDA > 4.0, p < 0.001), which were enriched in the PBS group, were effectively cleared after treatment, while beneficial bacteria such as *Lachnospiraceae* and *Oscillospiraceae* (Figure 5g,h). The *L. reuteri*@ZGM treatment demonstrated exceptional ability to reshape the gut bacterial microbiota in a DSS‐induced colitis mouse model. Its effects were not only reflected in the restoration of microbial diversity but also extended to the targeted enrichment of key functional bacterial groups and the suppression of pathogens.

### 2.7 *L. Reuteri*@ZGM Treatment Attenuates Chronic Colitis and Intestinal Barrier Dysfunction in *IL‐10* ^−/−^ Mice

**FIGURE 6:**
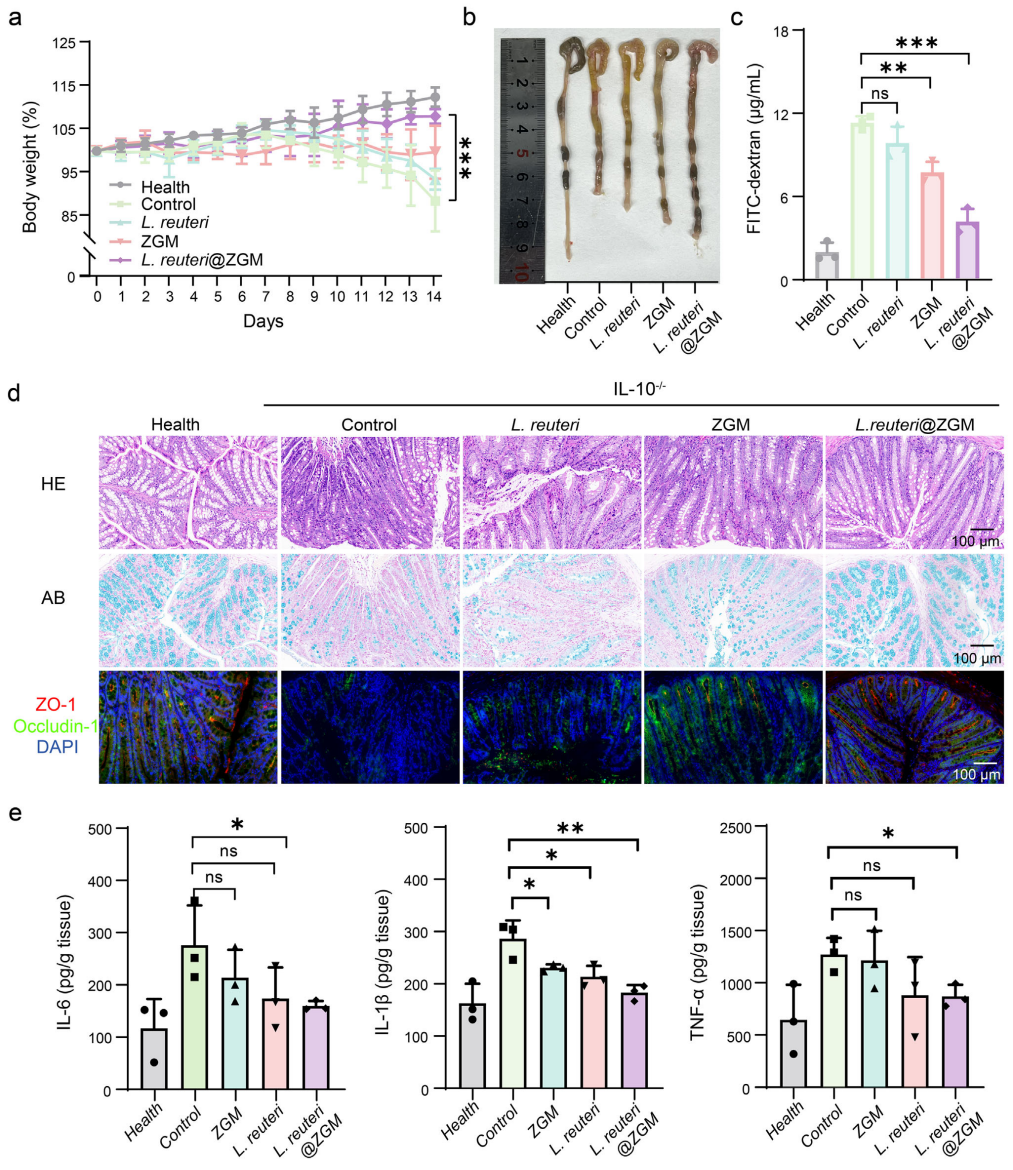
Effect of *L. reuteri*@ZGM on chronic colitis in *IL‐10* ^−/−^ mice. a) Change in body weight of mice in each group over a 14‐day period (n = 5). b) Representative colonic tissue images of mice in each group at day 14 (n = 5). c) On day 14, the intestinal barrier function was assessed in vivo in each group of mice by oral administration of 4 kDa FITC‐dextran, and fluorescence intensity in serum was measured 4 h post‐administration. d) Representative images of HE, AB, and ZO‐1/Occludin‐1 staining in colon tissue from mice in each group. Scale bar, 100 µm. e) Pro and anti‐inflammatory cytokines levels in colonic tissue were analyzed at day 14 (n = 3). Data represent geometric means ± SEM, with *p* values determined by Student's *t‐test*. *p < 0.05, **p < 0.01, and ***p < 0.001.

Building on the superior efficacy of *L. reuteri*@ZGM in DSS‐induced colitis, we further evaluated the therapeutic potential of *L. reuteri*@ZGM in the *IL‐10* ^−/−^ spontaneous colitis model. *IL‐10* ^−/−^ mice, known for developing chronic colitis, exhibited weight loss, decreased survival, increased inflammatory factors levels, and notable intestinal inflammation and phenotype abnormalities [34]. As expected, *L. reuteri*@ZGM treatment effectively attenuated weight loss, reduced the DAI, and decreased spleen index (Figure 6a,b **;** Figure S19–S21). The colon's length and morphology serve as key indicators of colitis severity. In *IL‐10* ^−/−^ mice, the colon wall was thickened, the intestinal body was swollen, the color was pale, the contents were thick and diffused, and the feces were irregular in shape. *L.reuteri*@ZGM treatment resulted in significant improvements, including increased colon length, thinning of the intestinal wall, and regular stool shape, similar to the healthy group. Additionally, *L. reuteri@ZGM* treatment significantly reduced intestinal permeability in *IL‐10* ^−/−^ mice, as evidenced by a lower systemic exposure to FITC‐glucan after oral administration compared to controls (Figure 6c). Notably, after *L. reuteri*@ZGM treatment, the number of goblet cells and the thickness of the mucosal layer in the colon were significantly increased, while the expression levels of tight junction proteins (ZO‐1 and Occludin‐1) in colon tissue were also upregulated, suggesting that *L. reuteri*@ZGM helped restore intestinal barrier integrity. Histological analysis using HE staining revealed severe inflammatory cell infiltration and crypt damage in the colon of untreated *IL‐10* ^−/−^ mice, while *L. reuteri*@ZGM treatment significantly reduced colonic inflammation and inflammatory cell aggregation (Figure 6d; Figure S22). Furthermore, *L. reuteri*@ZGM treatment significantly decreased the expression of pro‐inflammatory cytokines (TNF‐α, IL‐1β, and IL‐6) in the colon compared to untreated *IL‐10* ^−/−^ mice (Figure 6e). The treatment also reduced elevated ROS levels in the colonic tissues of *IL‐10* ^−/−^ mice and decreased apoptosis in the colonic epithelial cells (Figure S23). Together, these findings demonstrate that *L. reuteri*@ZGM treatment attenuates colonic inflammation, restores tight junction integrity, and improves the intestinal barrier function in the *IL‐10* ^−/−^ spontaneous colitis model.

## 3 Conclusion

Various delivery technologies—such as polymer coatings, liposomes, hydrogels, and biofilms—have been explored to enhance probiotic protection and delivery, few are tailored to dynamically respond to the pathological microenvironment of IBD [43, 44, 45]. In this study, we addressed this unmet need by constructing a multifunctional delivery platform for zinc‐crosslinked glycyrrhizic acid microgels (ZGM) based on the clinical anti‐inflammatory agent Licorzinc. Natural products such as glycyrrhizin and its derivatives have long been recognized for their broad‐spectrum bioactivities—including anti‐inflammatory, and antioxidant effects—with low systemic toxicity [46, 47]. By self‐assembling GA with zinc ions, we developed a gel matrix that combine the functions of probiotic protection carrier and drug therapy (Scheme 1) [48]. The resulting *L. reuteri*@ZGM system demonstrated multiple synergistic functions: it enhanced bacterial resistance to gastric acid by over 23‐fold, improved colonization efficiency by over 37‐fold, and scavenged ROS at sites of inflammation. The system also promoted mucosal adhesion, supporting robust probiotic survival and long‐term retention in the gut.

In two distinct preclinical models—DSS‐induced colitis and *IL‐10* ^−^^/^^−^ spontaneous colitis, *L. reuteri*@ZGM significantly reduced intestinal inflammation, suppressed oxidative stress, protected epithelial cells from apoptosis, and restored tight junction protein expression, thereby improving intestinal barrier function. Moreover, 16S rRNA sequencing revealed that *L. reuteri*@ZGM rebalanced gut microbiota composition by increasing microbial richness and diversity. Notably, the treatment elevated the abundance of beneficial commensals such as *Lachnospiraceae* and *Muribaculaceae*, while suppressing pro‐inflammatory taxa like *Enterobacteriaceae*, demonstrating its capacity to remodel a dysbiotic microbiota in a disease‐relevant context. However, direct evidence of gut microbiota modulation in the IL‐10^−^^/^^−^ model is lacking, which represents a limitation of this study. Given the sensitivity of gut microbiota composition to environmental factors (e.g., diet and housing conditions), cross‐study comparisons may not be sufficient. Future studies incorporating parallel microbiome analyses in DSS‐treated and IL‐10^−^^/^^−^ models will be necessary to establish whether *L. reuteri*@ZGM consistently reshapes the microbiota across disease contexts and to strengthen mechanistic insights into host–microbiome interactions.

Although our results are compelling, this study is limited to mouse models, which differ from humans in intestinal physiology, immunity, and microbiota composition, potentially affecting clinical predictability. Long‐term biocompatibility of ZGM, including the risk of zinc accumulation and chronic intestinal effects, requires thorough evaluation. Compared with conventional probiotic delivery systems such as polymer coatings and liposomes, ZGM integrates microenvironmental modulation with therapeutic functions, leveraging the clinically established safety of zinc glycyrrhizate. Nonetheless, the feasibility of large‐scale production and clinical translation requires further optimization and validation.

In summary, the ZGM platform demonstrates promising potential for synergistic probiotic therapy. Its translational advancement will require systematic assessment in advanced preclinical models and long‐term safety studies.

## 4 Experimental Section

### 4.1 Materials and Strains

The probiotic *L. reuteri* was obtained from BeNa Chuang Lian Biotechnology Research Institute (Beijing, China). Glycyrrhizic acid was obtained from Nanjing Jingzhu Bio‐Technology Co., Ltd. Anhydrous zinc sulfate (ZnSO_4_), 1, 1‐diphenyl‐2‐picratehydrazyl (DPPH), and bile salt were purchased from Mackin (Shanghai, China). Lipopolysaccharide (LPS) was provided by Solarbio (Beijing, China). Citric acid was purchased from Kermel (Tianjin, China). Wheat germ agglutinin conjugated with FITC (FITC‐WGA) was purchased from Maokang (Shanghai, China). Gastric juice was bought from Shanghai Yuanye Bio‐Technology Co., Ltd. Dextran sulfate sodium (DSS, molecular weight, 36–50 kDa) was obtained from MP Biomedicals (California, USA). IL‐6, IL‐10, IL‐1β, TNF‐α, and TGF‐β enzyme‐linked immunosorbent assay (ELISA) kits were obtained from Shanghai Enzyme‐linked Biotechnology Co., Ltd.

### 4.2 The Preparation of ZG Gel

ZG gel was prepared by mixing 3% GA solution with 0.03% ZnSO_4_ solution by simple heating and cooling. First, 0.3 g GA was dissolved in 10 mL water, and NaOH was added to adjust the solution to pH = 7.0. 0.003 g ZnSO_4_ was added to the solution and mixed by stirring. This was followed by heating in a water bath at 80°C for 30 min and cooling to obtain 10 mL of 3% ZG gel.

### 4.3 Characterization of ZG Gel

Scanning electron microscopy (SEM, TESCAN) and transmission electron microscopy (TEM, JEM) were used to observe the morphology of ZG gel. Fourier transform infrared (FT‐IR) spectra of ZG gel was obtained using an infrared spectrophotometer (Nicolet). The chemical composition of ZG gel was analyzed by X‐ray photoelectron spectroscopy (XPS, Thermo). Rheological analysis of the ZG gel was performed using MCR‐92 rheometer (Anton Paar, Austria). The zeta potential of the ZG gel was analyzed by dynamic light scattering (DLS, Malvern nano 25).

### 4.4 Determination of Antioxidant Activity

DPPH and ABTS were selected to evaluate the ROS scavenging ability of ZG gel. Samples were mixed with 3 mL of DPPH or ABTS solution and allowed to react in the dark for 20 min. Then, the full wavenumber scanning curves were recorded, and the absorbance values at 517 and 734 nm were measured.

### 4.5 Anti‐Inflammatory Properties of ZG Gel

Mouse mononuclear‐macrophages (RAW264.7) were seeded in 6‐well plates at a density of 1 × l0 [6] cells per well and then pretreated either GA or ZG gel for 24 h, respectively. RAW264.7 cells were stimulated with LPS (1 µg mL^−1^, SMB00610, Sigma) for 48 h. The supernatants were collected, and the concentrations of IL‐6 and IL‐1β were determined by ELISA kit. In addition, the cell lysates were collected, and NF‐κB expression was examined by Western blotting.

### 4.6 Flow Cytometry Assay

RAW 264.7 were seeded into 6‐well plates and allowed to adhere overnight. To induce M1 polarization, cells were treated with LPS (1 µg mL^−1^). To induce M2 polarization, cells were treated with IL‐10 (20 ng mL^−1^, Peprotech). For drug intervention, cells were pretreated with either ZGM or ZG gel for 1 h, followed by stimulation with LPS or IL‐10 for 24 h, respectively. The control group received only culture medium. After 24 h of incubation, cells were harvested and washed twice with ice‐cold PBS for subsequent flow cytometry analysis. For flow cytometry, cells were resuspended in PBS containing 2% bovine serum albumin (BSA). For CD86 staining (surface marker), cells were incubated with PE‐conjugated anti‐mouse CD86 antibody (BioLegend) for 30 min at 4°C in the dark. For CD206 staining, cells were first fixed and permeabilized using a fixation/permeabilization kit (BioLegend) following the manufacturer's guidelines, then stained with Alexa Fluor 647 anti‐mouse CD206 antibody (BioLegend) for 30 min at 4°C in the dark. The fluorescence of the cells was analyzed using flow cytometry and FlowJo 10.8 software.

### 4.7 Preparation of ZGM or *L. Reuteri*@ZGM

To obtain emulsions containing GA, 5 mL of GA solution was obtained using the same method as described above, with or without 2.5 mL of probiotic precipitate (∼ 10^9^ CFUs mL^−1^). The mixture was stirred for an additional 15 min, then 5 mL of the oil phase was added to a final concentrat ion of 2 wt.% Tween 80. The w/o emulsions were produced using a mechanical stirrer at 170 rpm. Another emulsion containing ZnSO_4_ was prepared in the same manner as the first emulsion. After stirring at 300 rpm, 10 mL of ZnSO_4_ emulsion was dropped into 10 mL of ZG gel probiotic emulsion. After 15 min, microcapsules were made, diluted with acetic acid solution (pH = 5.5), stirred at 170 rpm for 15 min, and left for 1 h. The precipitate was collected by centrifugation at 3500 rpm as ZGM or *L. reuteri*@ZGM.

### 4.8 Properties of Encapsulated Probiotics

SEM was used to observe the ZGM morphology of the encapsulated probiotics. Colocalization of EcN‐GFP encapsulated by Congo red‐labeled ZGM was examined by CLSM.

### 4.9 Growth Curves of Encapsulated Probiotics

*L. reuteri* was harvested at the late logarithmic growth phase, a standard practice for probiotic preparation, and immediately encapsulated to preserve metabolic activity and survival potential. The probiotics were released by crushing *L. reuteri*@ZGM (1 mL) with magnetic stirring in the presence of 9 mL sodium citrate solution (5.0%). The probiotics were diluted with MRS broth to OD_600_ = 0.2 using free *L. reuteri* as a control and cultured in an incubator at 37°C with shaking at 200 rpm. OD_600_ values of probiotics were recorded at 1 h intervals using a microplate spectrophotometer (Molecular Devices).

### 4.10 In Vitro Protective Capacity Assay

Equal volumes of *L. reuteri* and *L. reuteri*@ZGM (1 × 10^9^ CFUs) were incubated serially in 1 mL of SGF and bile salts to mimic the harsh GI environment. The solution was centrifuged and resuspended in MRS broth and incubated at 37°C with gentle shaking. OD_600_ values were measured at 1‐h intervals and growth curves were plotted. In addition, *L. reuteri* and *L. reuteri*@ZGM (1 × 10^9^ CFUs) were resuspended in 2 mL SGF and incubated for 2 h at 200 rpm in a 37°C incubator. *L. reuteri* and *L. reuteri*@ZGM were resuspended in 2 mL bile salts and incubated for 6 h at 200 rpm in a 37°C incubator. 50 µL of each sample was taken at specific time points and spread on MRS agar medium. The cells were grown overnight in an incubator at 37°C, and colonies were counted. At the same time, *L. reuteri* was stained using DAPI and PI and visualized by CLSM.

### 4.11 Survival of Probiotics in Mice

8‐week‐old BALB/c female mice in each group received 200 µL of *L. reuteri* and *L. reuteri*@ZGM containing 1 × 10^9^ CFUs orally. The stomach, small intestine, colon, and cecum of mice were harvested at 4, 48, and 96 h to evaluate *L. reuteri* adhesion and colonization in the GI tract. 20 µL of homogenized tissue was spread on MRS agar medium. The plates were incubated for 12 h at 37°C and counted.

### 4.12 Distribution of Probiotics in the GI Tract

In vivo fluorescence imaging system (IVIS SPECTRUM CT) was used to observe the distribution of GI tract after oral probiotics. 42 mice were randomly divided into EcN‐mCherry group and EcN‐mCherry@ZGM group. Each mouse was gavaged with 200 µL (1 × 10^9^ CFUs mL^−1^) of bacterial suspension. Three rats in each group were sacrificed at 1, 2, 4, 6, 12, 24, and 48 h, respectively, and the intestines were taken for imaging.

### 4.13 Long‐Term Adhesion of Probiotics In Vivo

8‐week‐old BALB/c mice were randomly divided into 3 groups after one week of adaptive feeding. Mice were gavaged with 200 µL of EcN‐mCherry, EcN‐mCherry@ZGM (1 × 10^10^ CFUs mL^−1^), and PBS. The above mice were sacrificed on days 3 and 6 after gavage to obtain colons and observed using CLSM.

### 4.14 3D Images of Mucus Adhesion

For 3D mucus adhesion observation, rat colons were cut into 4 cm lengths, and sections were closed with medical sutures. 100 µL FITC‐WGA (10 µg mL^−1^) was added for staining. After colon tissue was removed, 200 µL of bacterial suspension containing EcN‐mCherry or EcN‐mCherry@ZGM (1 × 10^10^ CFUs mL^−1^) was slowly added to the mucus surface. Finally, after 1 h at 37°C, the distribution of bacteria in the mucosal layer was determined by CLSM.

### 4.15 Animals

Female BALB/c mice (7–8 weeks old) were selected based on established practices in DSS‐induced colitis models. Female BALB/c mice were provided by GemPharmatech LLC. All animal experiments were approved by the Committee for Experimental Animals Welfare and Ethics of Hunan University (HNU‐IACUC‐2021‐102).

### 4.16 Mouse DSS‐Induced Colitis Model

8‐week‐old female BALB/c mice were fed with 3% DSS as previously described for 1 week. Mice were randomly divided into 5 groups and received ZGM, *L. reuteri* (2 × 10^9^ CFUs day^−1^), *L. reuteri*@ZGM, or PBS orally for 5 days. Changes in body weight were assessed daily during the 12 days trial. In order to confirm the successful establishment of colitis model, the changes of DAI were recorded every day during the establishment of colitis model. On the last day, mice were sacrificed and blood was collected for H&E and immunofluorescence staining of the whole colon. In addition, feces were collected for gut microbiota analysis.

### 4.17 Histopathological Analysis

Colons were fixed in tissue fixative, embedded in paraffin, and stained with H&E according to standard procedures. Finally, the staining results were observed by fluorescence microscope.

### 4.18 In Vivo Immunofluorescence Imaging

Briefly, collected colon tissues were fixed in 4% paraformaldehyde, embedded in paraffin, and sectioned into 4 µm slices. The sections were deparaffinized with xylene, rehydrated through a graded ethanol series, and subjected to antigen retrieval by microwaving in 10 mM sodium citrate buffer (pH 6.0). After cooling and washing with PBS, the sections were blocked with immunostaining blocking solution for 30 min at room temperature. Subsequently, the sections were incubated overnight at 4°C with a mixture of two directly fluorophore‐conjugated primary antibodies: anti‐ZO‐1 antibody directly conjugated to Alexa Fluor 488 (5 µg mL^−1^; Catalog # GB151981, Servicebio) and anti‐Occludin antibody directly conjugated to Alexa Fluor 594 (1 µg mL^−1^; Catalog # GB111401, Servicebio). Following overnight incubation, the sections were thoroughly washed with PBS to remove unbound antibodies. Cell nuclei were then counterstained with Hoechst solution. After a final wash, the slides were mounted with anti‐fade mounting medium. Fluorescence images were acquired using a CLSM. ROS and apoptosis were performed: Tissue sections were stained with dihydroethidium (DHE) solution according to the manufacturer's protocol. Apoptotic cells were detected using the One‐step TUNEL Apoptosis Detection Kit (Catalog # GB22403, Servicebio), following the manufacturer's instructions.

### 4.19 In Vivo Enzyme‐Linked Immunosorbent Assay (ELISA) Analysis

To determine cytokine levels in colon tissue, colon tissue sections were homogenized at 4°C using PBS (mass/volume ratio of 1:10). Each sample was centrifuged at 10,000 × g for 10 min at 4°C. An enzyme‐linked immunosorbent assay (ELISA) kit (Shanghai Enzyme‐linked Biotechnology Co., Ltd.) was used to detect the levels of IL‐6, IL‐10, IL‐1β, TGF‐β, and TNF‐α in the supernatant of the colon tissue. According to the manufacturer's instructions, each kit contained specific monoclonal detection antibodies, capture antibodies, and HRP‐labeled secondary antibodies. All procedures were repeated three times according to the manufacturer's protocol, and concentration values were calculated using a standard curve.

### 4.20 Measurement of Intestinal Permeability In Vivo

Intestinal permeability assays were performed using 4 kDa FITC‐glucan (Beyotime) as previously described. After 4 h of food and water deprivation, mice were orally administered 0.6 mg g^−1^ body weight FITC‐glucan. Mice were sacrificed, and serum samples were obtained for determination of FITC fluorescence intensity.

### 4.21 16S Sequencing and Microbiome Analysis

After treating the colitis mouse model, collect 2–3 fecal samples (50‐100 mg) from each mouse, rapidly freeze them in liquid nitrogen, and store them at ‐80°C for later use. DNA was extracted using the DP328 DNA Extraction Kit (TIANGEN), with the 16S V4 region as the amplification target (Forward primer 515F: 5′‐GTGCCAGCMGCCGCGGTAA‐3′, Reverse primer 806R: 5′‐GGACTACHVGGGTWTCTAAT‐3′). After quality control of the amplification, mix and purify the PCR products. PCR conditions: Denature for 1 min at 98°C, followed by 30 cycles, each consisting of 10 s of denaturation at 98°C, 30 s of annealing at 50°C, and 30 s of extension at 72°C, followed by a final extension of 5 min at 72°C. The PCR products were purified and pooled to construct sequencing libraries. All libraries were quantified using Qubit and qPCR. After the libraries were verified asvalid, 16S rRNA sequencing was performed on the Illumina NovaSeq 6000 platform using the PE250 mode. Sample data were separated from the raw sequencing data based on the barcode sequences and PCR primer sequences. After trimming the barcode and primer sequences, FLASH (Version 1.2.11) was used to assemble the reads for each sample, yielding the assembled sequences as Raw Tags. Subsequently, Cutadapt software was used to match the reverse primer sequences and trim the remaining sequences to prevent interference with subsequent analyses. The assembled Raw Tags were rigorously filtered using the fastp software (Version 0.23.1) to obtain Clean Tags. The Tags obtained after the above processing were then processed to remove chimeric sequences. The Tags sequences were compared against species annotation databases (Silva database https://www.arb‐silva.de/ for 16S/18S, Unite database https://unite.ut.ee/ for ITS) to detect chimeric sequences, and the chimeric sequences were ultimately removed to obtain the Effective Tags. For the resulting Effective Tags, the DADA2module to perform noise reduction, thereby obtaining the final Amplicon Sequence Variants (ASVs) and feature tables. Species annotation was performed using QIIME2 with the Silva138.1 database. Finally, the data from each sample were normalized using the sample with the smallest data volume as the standard; all subsequent analyses were based on the normalized data. We performed abundance analysis, α‐diversity calculations, Venn diagrams, and petal plots on the ASVs to obtain information on species diversity and abundance within samples, as well as ASVs shared among or unique to different samples or groups. Additionally, through dimension‐reduction analyses such as PCoA, PCA, and NMDS, as well as the visualization of sample clustering trees, we investigated differences in community structure among different samples or groups. For beta diversity analysis, four metrics—weighted Unifrac distance, unweighted Unifrac distance, Jaccard distance, and Bray‐Curtis distance—were selected to measure differences between two samples. We used the Adonis and Anosim methods to analyze whether differences in community structure between groups were statistically significant; through statistical analysis, we specifically identified species with significant differences in abundance between groups and determined the enrichment patterns of these differential species within different groups. To further explore differences in community structure among grouped samples, statistical analysis methods such as LEfSe (*p* < 0.001 and LDA effect size > 4) were employed to test for significant differences in the microbial communities significantly enriched in different groups.

### 4.22 *IL‐10^−/−^* Model of Spontaneous Colitis

*IL‐10* ^−/−^ mice (10 weeks of age) were obtained from GemPharmatech LLC. Untreated *IL‐10* ^−/−^ mice (control) spontaneously developed colitis. For treatment groups, *IL‐10* ^−/−^ mice were orally administered ZGM, *L. reuteri* (1 × 10^8^ CFUs day^−1^), *L. reuteri*@ZGM, or PBS daily from the age of 10 weeks until the end of the experiment.

### 4.23 Statistical Analysis

All data are presented as mean ± SD. n = 3 for in vitro experiments and quantification analysis; n = 5 or 6 for in vivo experiments. For statistical analysis, GraphPad Prism v.8.0.2 (GraphPad Software) was used. P values are calculated using a one‐tailed Student's *t‐test*. *p < 0.05, **p < 0.01, ***p < 0.001 are used to indicate statistical significance.

## Author Contributions

**Mengyun Peng**: conceptualization, methodology, data curation, supervision, Writing – original draft, visualization, project administration, formal analysis. **Xiaochun Chen**: validation, formal analysis, visualization, investigation, methodology. **Wei Li**: validation, visualization. **Shu Tang**: validation. **Jun Zhou**: resources. **Xia Wu**: resources, project administration. **Dan Yuan**: funding acquisition, resources, project administration. **Zhongyan Shan**: resources, writing – review and editing, funding acquisition. **Junfeng Shi**: writing – review and editing, project administration, funding acquisition, resources, conceptualization, methodology, data curation, supervision.

## Funding

This work was partially supported by the National Natural Science Foundation of China (32401127 to D.Y.), National Youth Talent Support Program (202309460011), Natural Science Foundation of Hunan (2024JJ5072 to D.Y., 2024JJ6084 to S.Z.Y.), The Key Project of Hunan Provincial Education Department (22A0020 to D.Y.), Natural Science Foundation of Guangdong (2024A1515011057, 2025A1515012954), Science and Technology and Development Foundation of Shenzhen (JCYJ20230807122008016), the Science and Technology Development Fund, Macau SAR (file no. 005/2023/SKL), supported by Hunan Provincial Innovation Foundation For Postgraduate (CX20250492 to M.Y.P.).

## Conflicts of Interest

The author declares no conflicts of interest.

## Supporting information




**Supporting File**: advs77674‐sup‐0001‐SuppMat.docx.

## Data Availability

The 16S rDNA sequencing data generated in this study have been deposited in the National Center for Biotechnology Information (NCBI) under accession number PRJNA1402698. All other data that support the findings of this study are available in the supplementary material of this article.
