# Supplementary material for: Multifunctional Zinc Glycyrrhizinate‐Integrated Gel for Enhanced Probiotic Delivery and Alleviation of Acute and Spontaneous Colitis
Source: Adv Sci (Weinh). 2026 Sep 13:e77674. Online ahead of print. doi: 10.1002/advs.77674 (PMC13572826; doi:10.1002/advs.77674)
Supplement: Supplementary file 1 [file ADVS-9999-e77674-s001.docx]

Supporting Information

Multifunctional Zinc Glycyrrhizinate-Integrated Gel for Enhanced Probiotic Delivery and Alleviation of Acute and Spontaneous Colitis

Mengyun Peng, Xiaochun Chen, Wei Li, Shu Tang, Jun Zhou, Xia Wu, Dan Yuan, Zhongyan Shan*, Junfeng Shi*

**Figure S1.** XPS high-resolution spectra of Zn 2p (A) and O 1s (B) signals for ZG gel. The Zn2p spectrum confirmed the valence state of Zn is +2. The O1s spectrum was deconvoluted to several peaks at 534.4 eV indicating the formation of Zn-O metal-ligand bonds, confirming the existence of metal-ligand bonds between -CO_3_- groups and Zn^2+^ ions.

**Figure S2.** Zeta potential measurement of the GA and ZG gel. The same concentration of GA powder and ZG gel lyophilized powder were used to measure the zeta potential by dynamic lights scattering (DLS).

**Figure S3.** Rheological measurement of ZG gel. (A) Frequency sweep of 1.0 wt% ZG gel showing the storage modulus (G') and loss modulus (G") as a function of angular frequency. (B) Strain sweep of ZG gel with strain ranging from 0.1% to 1000% at a constant angular frequency of 6 rad/s.

**Figure S4.** Biocompatibility Assessment of ZG Gel. ZG gel at different concentrations was added to the culture medium. After incubating Caco-2 cells at 37°C for 24 hours, cell viability was assessed using a live/dead staining assay (A) and MTT assay (B). Data are presented as means ± SD (n = 3). **P* < 0.05, ***P* < 0.01, ****P* < 0.001, determined by Student’s *t-test*.

**Figure S5.** Stability evaluation of ZG gel under different pH conditions over time. ZG gel was incubated in buffer solutions with varying pH values (pH 1.2, 6.8, and 7.4) at room temperature, and its physical state was monitored over time to assess the stability.

**Figure S6.** Flow cytometric analysis of CD86 and CD206 expression on RAW 264.7 macrophages under different treatments. Relative expression of CD86 and CD206 in RAW 264.7 cells exposed to LPS, LPS + GA and LPS + ZG gel. Data are presented as means ± SD (n = 3). **P* < 0.05, ***P* < 0.01, ****P* < 0.001, determined by Student’s *t-test*.

**Figure S7.** Quantitative analysis of NF-κB expression level in RAW 264.7 after different treatment. Relative expression of NF-κB in RAW 264.7 cells exposed to LPS, LPS + GA and LPS + ZG gel. Data are presented as means ± SD (n = 3). **P* < 0.05, ***P* < 0.01, ****P* < 0.001, determined by Student’s *t-test*.

**Figure S8.** Photographs illustrating the adhesive properties of ZG gel on porcine skin.

**Figure S9.** Size distribution of microgel ZGM.

**Figure S10.** Quantification of bacterial colonization in the gut over time. Mice were orally administrated with 1 × 10^9^ CFUs of *L. reuteri* or *L. reuteri*@ZGM and then executed at 4, 48, and 96 hours post-administration. Gut contents were collected, serially diluted with PBS, and 20 μL of each dilution was spread on the MRS agar plates with kanamycin. Then the colonies were counted after incubation overnight at 37°C. Data are presented as means ± SD (n = 3). **P* < 0.05, ***P* < 0.01, ****P* < 0.001 determined by Student’s *t-test*.

**Figure S11.** Quantitative analysis of the distribution of EcN-mCherry and EcN-mCherry@ZGM in the gastrointestinal tract within 48 hours after oral administration. Data are presented as means ± SD (n = 3). **P* < 0.05, ***P* < 0.01, ****P* < 0.001 determined by Student’s *t-test*.

**Figure S12.** Biodistribution of EcN-mCherry, EcN-mCherry@ZGM in major organs (heart, liver, spleen, lung and kidney) within 48 hours after oral administration by intestinal transit assay.

**Figure S13.** (A-B) Bacterial viability of *L. reuteri* or *L. reuteri*@ZGM with or without H_2_O_2_ (500 μM) treatment, assessed by CCK-8 measuring. Data are presented as means ± SD (n = 3). **P* < 0.05, ***P* < 0.01, ****P* < 0.001 determined by Student’s *t-test*.

**Figure S14.** Representative images of AB/APS and TUNEL staining in the colonic tissues from each group. Scale bar: 200 μm.

**Figure S15.** Histology scoring of colon tissues based on H&E staining. Data are presented as means ± SD (n = 6). **P* < 0.05, ***P* < 0.01, ****P* < 0.001 determined by Student’s *t-test*.

Figure S16. (a) INOS-positive and CD206-positive cells in colonic tissues from each group. Scale bar: 200 μm. (b) Quantitative analysis of the relative area coverage of INOS-positive and CD206-positive signals in each group. Data are presented as means ± SD (n = 6). **P* < 0.05, ***P* < 0.01, ****P* < 0.001 determined by Student’s *t-test*.

**Figure S17.** H&E staining of major organs (the intestine, heart, liver, spleen, lung and kidney) collected from mice on day 13 following oral administration of 1 × 10^9^ CFUs of *L. reuteri*, *L. reuteri* @ZGM, or ZGM. PBS-treated mice served as control group. Scale bar, 200 μm.

**Figure S18.** Venn diagram showing the similarity and overlap of OTU composition among different treatment groups.

**Figure S19.** DAI scores of mice across different groups. Data are presented as means ± SD (n = 5). **P* < 0.05, ***P* < 0.01, ****P* < 0.001 determined by Student’s *t-test*.

**Figure S20.** Colon length measurements in each group of mice. Data are presented as means ± SD (n = 5). **P* < 0.05, ***P* < 0.01, ****P* < 0.001 determined by Student’s *t-test*.

**Figure S21.** Spleen index of mice in each group measured on day 15. Data are presented as means ± SD (n = 5). **P* < 0.05, ***P* < 0.01, ****P* < 0.001 determined by Student’s *t-test*.

**Figure S22.** Histology scoring of colon tissues based on H&E staining. Data are presented as means ± SD (n = 5). **P* < 0.05, ***P* < 0.01, ****P* < 0.001 determined by Student’s *t-test*.

**Figure S23.** Representative images of AB/APS and TUNEL staining in the colon of mice in each group. Scale bar: 100 μm.

**Table S1**: DAI parameters and their associated scoring schemes. The DAI score is the sum of the scores for body weight, stool consistency, and blood in stool.

| DAI parameters and their associated scoring schemes | | | |
| --- | --- | --- | --- |
| Score | Weight loss (%) | Stool consistency | Blood in stool |
| 0 | None | Normal | Normal |
| 1 | 1-5 | Slightly loose stool | Small presence of blood |
| 2 | 5-10 | loose stool | Significant presence of blood |
| 3 | 10-15 | Diarrhea | Gross blood |
| 4 | >15 |  |  |
